# Uncovering Ultrastructural Defences in *Daphnia magna* – An Interdisciplinary Approach to Assess the Predator-Induced Fortification of the Carapace

**DOI:** 10.1371/journal.pone.0067856

**Published:** 2013-06-12

**Authors:** Max Rabus, Thomas Söllradl, Hauke Clausen-Schaumann, Christian Laforsch

**Affiliations:** 1 Department of Biology II, Ludwig-Maximilians-University Munich, Germany; 2 Department of Animal Ecology I, University of Bayreuth, Germany; 3 Department of Precision, and Micro-Engineering, Engineering Physics, University of Applied Sciences Munich, Germany; 4 Center for NanoScience, Ludwig-Maximilians-University Munich, Germany; 5 Geo-Bio Center, Ludwig-Maximilians-University Munich, Germany; University of Western Ontario, Canada

## Abstract

The development of structural defences, such as the fortification of shells or exoskeletons, is a widespread strategy to reduce predator attack efficiency. In unpredictable environments these defences may be more pronounced in the presence of a predator. The cladoceran *Daphnia* *magna* (Crustacea: Branchiopoda: Cladocera) has been shown to develop a bulky morphotype as an effective inducible morphological defence against the predatory tadpole shrimp *Triopscancriformis* (Crustacea: Branchiopoda: Notostraca). Mediated by kairomones, the daphnids express an increased body length, width and an elongated tail spine. Here we examined whether these large scale morphological defences are accompanied by additional ultrastructural defences, i.e. a fortification of the exoskeleton. We employed atomic force microscopy (AFM) based nanoindentation experiments to assess the cuticle hardness along with tapping mode AFM imaging to visualise the surface morphology for predator exposed and non-predator exposed daphnids. We used semi-thin sections of the carapace to measure the cuticle thickness, and finally, we used fluorescence microscopy to analyse the diameter of the pillars connecting the two carapace layers. We found that *D* *. magna* indeed expresses ultrastructural defences against *Triops* predation. The cuticle in predator exposed individuals is approximately five times harder and two times thicker than in control daphnids. Moreover, the pillar diameter is significantly increased in predator exposed daphnids. These predator-cue induced changes in the carapace architecture should provide effective protection against being crushed by the predator’s mouthparts and may add to the protective effect of bulkiness. This study highlights the potential of interdisciplinary studies to uncover new and relevant aspects even in extensively studied fields of research.

## Introduction

Predation is one of the major drivers of natural selection [1,2]. To avoid predation, prey species have evolved a variety of defence mechanisms. Such defences may be constitutive, if there is a unpredictable but permanent threat of predation, or they may be inducible, i.e. they are only expressed when there is an actual risk of predation [3]. There are four prerequisites for the evolution of inducible defences: An unpredictable, varying, but sometimes strong predation pressure; the existence of a reliable signal indicating the threat; the expression of a defence that offers effective protection; and finally, the defence should incur costs that outweigh the benefits in times when the predator is not present [3]. Inducible defences, which include behavioural, life history and morphological alterations, are a widespread strategy across a broad range of taxa, including bacteria, plants, rotifers, molluscs, crustaceans, insects and vertebrates [4–6]. Inducible morphological defences such as long spines in rotifers [7], an altered shell morphology in barnacles [8] and a deeper body in crucian carps [9] are expressed to impede or prevent capture and ingestion by the predator [10]. Moreover, morphological defences include structural defences which offer protection from being crushed by the predator. For instance, marine and freshwater snail species increase the thickness of their shell in response to predatory crabs and fish, respectively [11,12]. Furthermore, studies in a variety of systems have demonstrated the effectiveness of such inducibly expressed protective armours (e.g. crab-induced increase in shell thickness in mussels [13], skeleton thickness in sea urchins [14], and fish induced increase in cuticle thickness in dragonfly larvae [15]).

In aquatic ecosystems, the expression of these inducible defences is often mediated by infochemicals released by the predator, so called kairomones, which the prey uses to assess the type and degree of predation risk [3,4]. Inducible defences are particularly well studied in cladocerans of the genus *Daphnia* , keystone grazers in aquatic ecosystems and important model organisms in biological research [17,18]. In *Daphnia* , a variety of inducible defences can be found, ranging from behavioural defences, e.g. diel vertical migration [19] and escape behaviour [20], to predator-induced life history shifts, e.g. an altered size and age at maturity [21,22], and morphology. Inducible morphological defences, which are predominantly expressed in response to gape-limited invertebrate predators, are thought to interfere with the feeding apparatus of the predator and thus increase the chance of escape from an attack [23]. Examples of morphological defences in *Daphnia* include: the Chaoborus-induced expression of large helmets in *D* *. ambigua* [24] and *D* *. cucullata* [25], the notonectid-induced formation of large dorsal crests in the *D* *. carinata* King complex [26,27], the elongation of the tail spine in fish-exposed *D* *. galeata* [28] and *D* *. lumholtzi* [29], the expression of a spine bearing, heart shaped lobe in the head region, the so called “crown of thorns” expressed by D. atkinsoni in response to the predatory tadpole shrimp Triops cancriformis [30], as well as a variety of other less conspicuous morphological defences in other *Daphnia* species, for instance, Chaoborus-induced small neckteeth in D. pulex [31–33].

The existence of ultrastructural defences in *Daphnia* , e.g. the fortification of the exoskeleton, has been widely discussed as an additional morphological defence that may accompany the prominent defensive structures [21,24,34]. However, only few studies could actually reveal such structural defence mechanisms. For instance, Dodson [35] could demonstrate that the predatory copepod Heterocope *septentrionalis* induces an increased cuticle thickness and strength in D. middendorffiana. Furthermore, it has been shown that Chaoborus-larvae induce a greater cuticle hardness in *D* *. cucullata* and D. pulex [36].

In the present study, we used the pond dwelling species *D* *. magna* , which has been shown to express inducible morphological defences against T. cancriformis. Thereby, induced individuals develop a bulky morphotype caused by an increased body length, body width, width of the shoulder shield and an elongated tail spine [37,38]. These defensive traits render the induced daphnids less susceptible to Triops-predation, presumably by impeding handling by the predator [38]. Here, we applied an interdisciplinary approach to investigate the Triops-induced ultrastructural defences in *D* *. magna* .

Unlike other high-resolution microscopy techniques, such as electron microscopy or scanning tunneling microscopy, atomic force microscopy (AFM) not only allows for high resolution imaging under physiological conditions [39], but it also provides the possibility to probe the mechanical properties of biological samples at the micrometer and the nanometre scale. In the force spectroscopy mode, AFM has been used to investigate the mechanical properties of covalent chemical bonds [40–42], single DNA molecules [43,44], proteins [45,46], tissue [47] or even living cells [48,49] in their physiological surroundings. Here we used AFM to record force maps on predator exposed and non-predator exposed *D* *. magna* . These nanoindentation experiments enabled the application of a defined force at multiple locations across the carapace and allowed us to measure the corresponding indentations of the cuticle as a function of the applied force. By using a modified Hertz model we were able to extract the corresponding hardness (Young’s Modulus) of the cuticle from this force vs. indentation curves. Generally speaking, at the same indentation force a lower indentation in predator exposed *Daphnia* compared to non-predator exposed ones indicates harder material properties,

Triops crushes its prey with its mandibles, thus increased cuticle hardness should be an effective defensive trait. In addition to cuticle hardness, we tested whether induced *D* *. magna* also increase the thickness of the cuticle, by the use of semithin sections of the carapace. Finally, we measured the diameter of the pillars connecting the outer and inner cuticle layer of the carapace which has been shown previously to be increased in induced *D* *. cucullata* [36]. For all measured traits we found considerable differences between predator exposed and non-predator exposed *D* *. magna* , i.e. cuticle hardness and thickness and the diameter of the pillars were increased in induced individuals.

## Materials and Methods

### Rearing of the experimental animals

**Figure 1:**
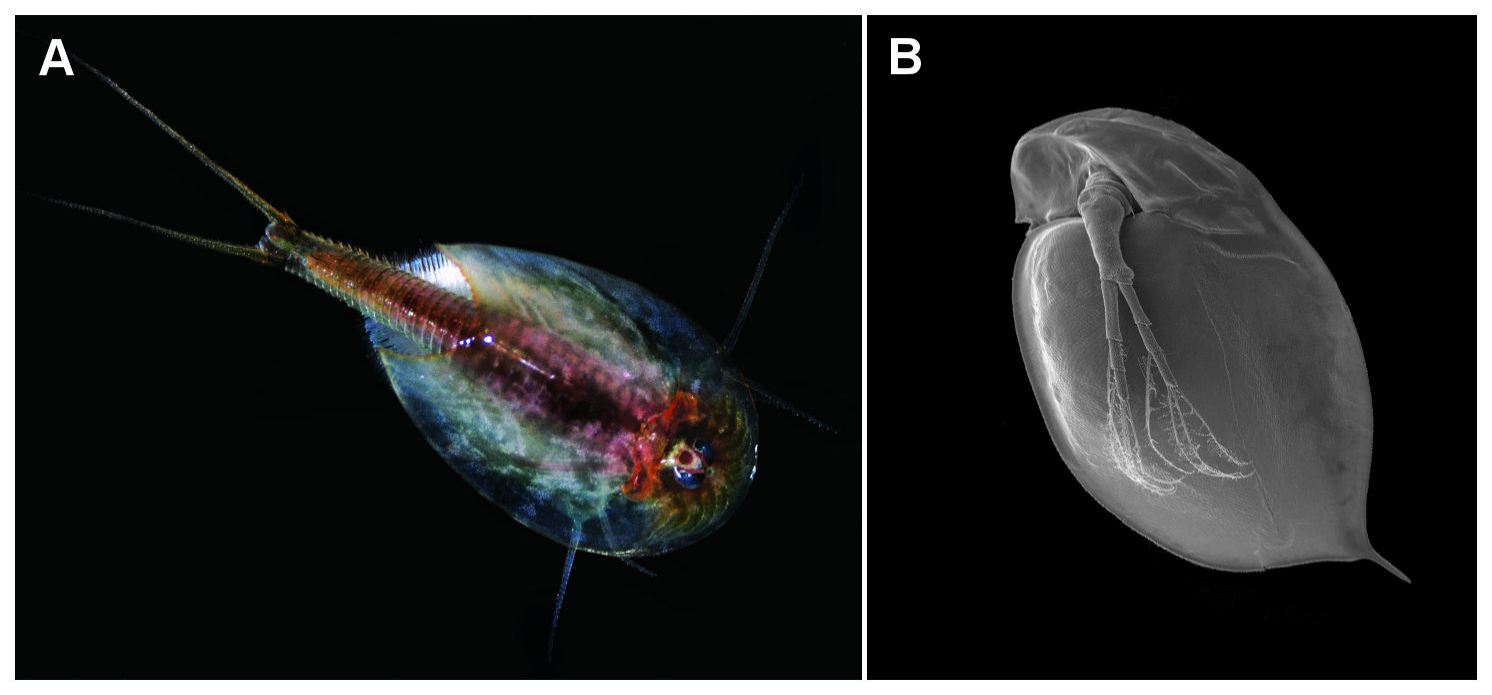
Morphological illustration of the experimental animals. A) Photograph of the predatory tadpole shrimp *Triopscancriformis* , one of the top predators in temporary ponds. *Triops* can reach a length of up to 10 centimetres, including the caudal furca. B) SEM picture of the cladoceran *Daphnia* *magna* , a common inhabitant of temporary waters in temperate regions. *D* *. magna* reaches a body length of up to 6 millimetres while the typical size at primiparity is between 2.5 and 3 millimetres.

 The experiment was conducted in a climate chamber at 20 ± 0.5 °C under fluorescent light with a day: night rhythm of 15: 9 hours. We used a single clonal line of *D* *. magna* (K_3_4J), which was isolated from a former fishpond near Munich, Germany, in 1998. No specific permits were required for the described field sampling, since it did not involve endangered or protected species and it was not conducted in a privately-owned area. This clone has previously been shown to express prominent inducible morphological defences against *T* *. cancriformis* [37] (for a morphological illustration of both, predator and prey, see Figure 1). As a predator, a laboratory cultured line of *T* *. cancriformis* , provided by Dr. E. Eder from the University of Vienna, was used (for a morphological illustration of both, predator and prey, see Figure 1). The experiment was carried out in 12L glass aquaria containing 10 litres of a semi-artificial medium based on ultrapure water, phosphate buffer and trace elements [37]. Each treatment, induction and control, was replicated three times. The bottoms of the aquaria were covered with a thin layer of sterilized sand (White Sun, Colorstone, Rudolstadt, Germany). The experiment was started by placing 60 randomly chosen, age synchronized, third clutch neonates of *D* *. magna* into each aquarium. Three small *T* *. cancriformis* with a body length of approximately 10 mm, measured from the anterior end of the carapace to the end of the abdomen, were placed into each aquarium destined for the induction treatment. *Triops* of this size were chosen as they trigger full induction in *D* *. magna* while remaining too small to effectively prey upon the daphnids. The daphnids were fed daily with 0.5 mg C/L of the green alga *Scenedesmus* *obliquus* . As a food source for *Triops*, we added 5 pellets of commercial fish food per day (JBL, Grana Discus, JBL GmbH & Co. KG, Neuhofen, Germany). The fish food was also added to the control aquaria to rule out any potential influence on the daphnids. To ensure good water quality, exuviae, faeces and fish food remnants were removed daily. Moreover, half of the medium was exchanged every five days.

 After releasing their offspring from the brood pouch, the adult daphnids were removed from the experimental units and the *Daphnia* density was adjusted to 60 daphnids per aquarium by randomly removing supernumerary neonates. This procedure was repeated three times until the third generation. Primiparous individuals of the third generation were then preserved for the analyses; the total duration of the induction experiment was approximately 27 days. For the analysis of cuticle hardness and pillar diameter, the daphnids were preserved in 70% ethanol. A 2.5% glutaraldehyde solution was used to preserve the daphnids destined for the analysis of cuticle thickness.

### Analysis of the carapace hardness using atomic force microscopy (AFM)

**Figure 2:**
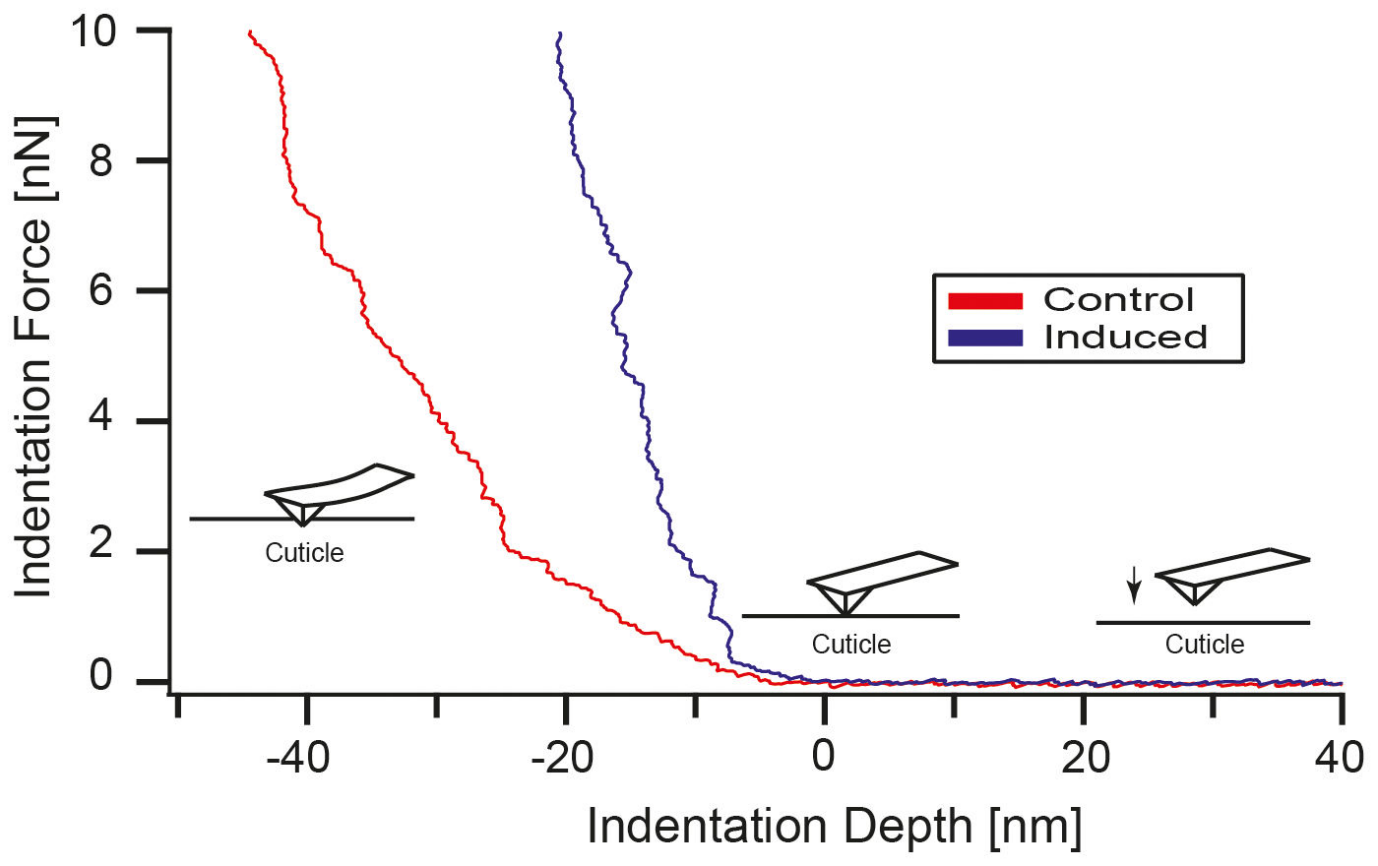
Qualitative comparison of force-distance curves obtained from predator- and non-predator exposed *D* *. magna* . Two characteristic force-distance curves taken on the cuticle of *D* *. magna* with (blue curve, predator exposed) and without (red curve, non-predator exposed) *T* *. cancriformis* present in culture. The cantilever is approached (from the right) to the carapace surface until it starts to be extended (middle part). This extension is continued until the maximum force of 10 nN is applied to the cuticle (left top). At the same indentation force, the non-predator exposed *Daphnia* shows a greater indentation than the predator exposed one, indicating softer material properties. For quantitative analysis of the carapace elasticity, a modified Hertz model is used to extract the Young’s modulus from each curve. Note that the maximum indentation does not exceed 60 nm, ensuring that only a small part of the approximately 2 µm thick cuticle is indented and that the local material properties of the cuticle are probed by the AFM.

AFM measurements were conducted using a commercial AFM (Nano Wizard, JPK Instruments AG, Berlin, Germany) with a 100 x 100 µm^2^ lateral scan range, equipped with a 100 µm z-extension stage (CellHesion, JPK Instruments AG, Berlin, Germany) resulting in a total z-range of 115 µm. A long distance microscope (Navitar Inc, Rochester NY, USA), connected to a CCD camera (The Imaging Source Europe GmbH, Bremen, Germany), was positioned over the AFM to precisely position the cantilever on the area of interest on the carapace. The force mapping experiments were carried out using sharpened silicon nitride cantilevers (OMCL-RC800PSA, Olympus Germany GmbH, Hamburg, Germany) with a nominal spring constant of 0,1 N/m and a pyramidal tip with a nominal tip radius of 15 nm. Prior to each experiment the actual cantilever spring constant was determined using the thermal noise method [50]. For each cantilever, three independent spring constant calibrations were carried out and the mean value was used for our experiments. All indentation experiments were performed in the semi-artificial medium described above. Prior to measurement, each ethanol-preserved *Daphnia* was mounted on a glass slide using a small droplet of commercial two-component adhesive (UHU plus sofortfest, Uhu GmbH & Co KG, Bühl, Germany). As soon as the glue bonded, the daphnids were covered with semi-artificial medium to prevent them from drying out. After positioning the cantilever above the area of interest, located in the centre of the carapace, a force map of a 75 x 75 µm^2^ area containing 5 x 5 measurement spots was probed. Thus, 25 force-indentation curves were obtained from each area of interest (Figure 2). Three different areas were evaluated on each animal. Five randomly chosen daphnids were analysed per replicate. The maximum indentation force was set to 10 nN. The resulting indentation depth of approximately 50 nm ensured that only the stiffness of the cuticle surface was probed.

The Young’s modulus (E) was extracted from the extend part of the force vs. indentation curves using a modified Hertz model for a four sided pyramidal indenter [51]:

$$F=0.7453\frac{E}{1-\nu^{2}}\delta^{2}tan\alpha$$ (Eq. 1)

Here *F* is the indentation force applied to the sample, *E* is the Young’s modulus, ν is the Poissons’s ratio (which was set to 0.5 for isotropic incompressible materials), δ the indentation and α the face angle of the pyramid (35° for our cantilever). The contact point was determined manually for each force indentation curve. Curves with large adhesion events in the retract part of the cycle were removed from the analysis. The data analysis was carried out using the JPK Data Processing Software (ver. 4.0.23, JPK Instruments, Germany).

### Surface topography analysis

**Figure 3:**
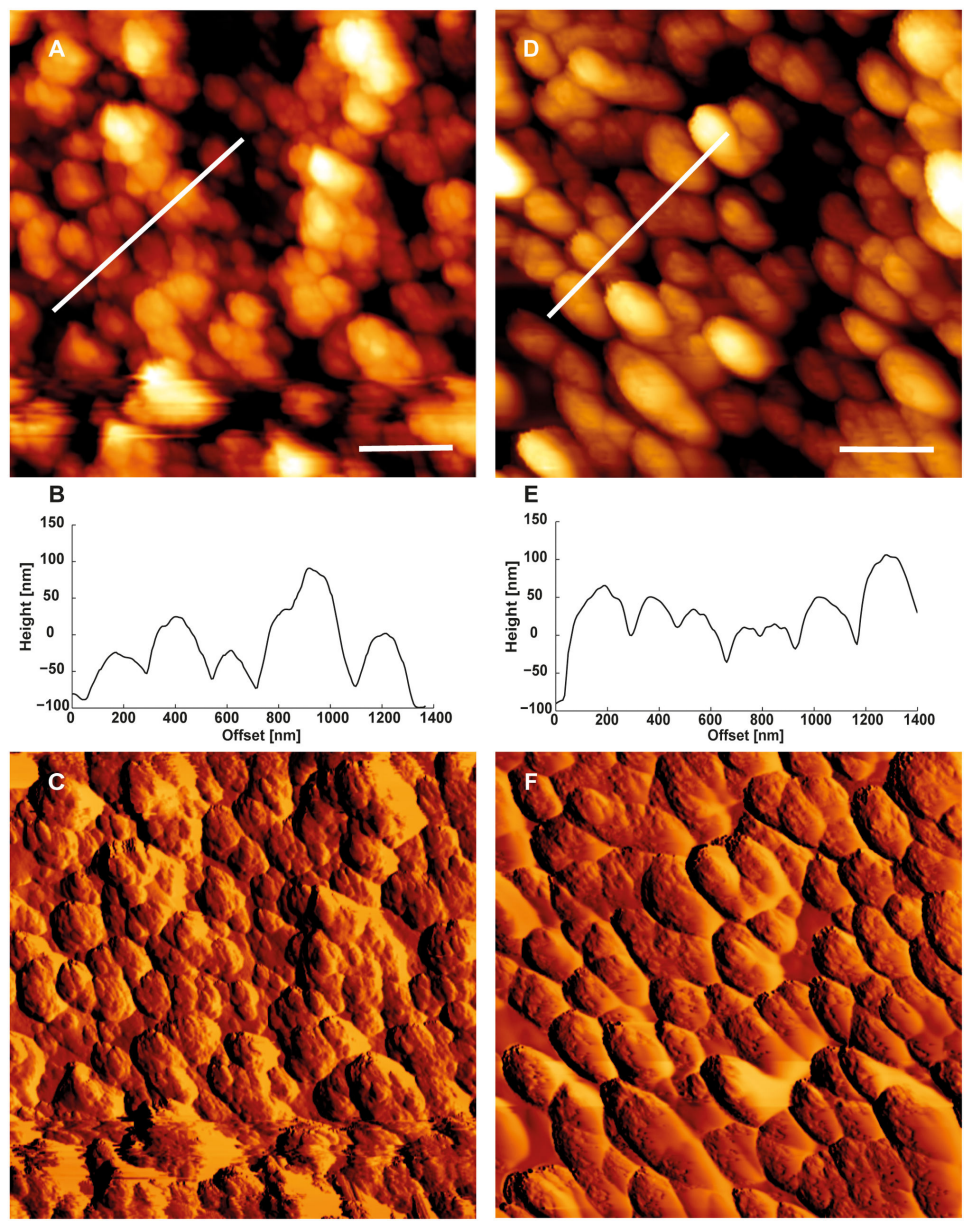
AFM images of the carapace surface for predator- and non-predator exposed *D* *. magna* . Topographical (A, D) and error images (C, F) taken on the carapace surface from non-predator exposed (A, C) and predator exposed (D, F) *D* *. magna* by AFM imaging. The white lines in the topographical images indicate the location taken to extract the height profile below (B, E). In addition to the found changes in the cuticle stiffness, AFM imaging indicated changes in the microstructure of the cuticle. While the cuticle surface of non-predator exposed *Daphnia* appears unstructured, the *Triops*-induced surface shows a highly organised microstructure. Scale bars represent 500 nm.

AFM imaging was performed in air on desiccated *D* *. magna* specimen. Samples were dried prior to analysis using a graded acetone series and hexamethyldisilazane [52]. High density carbon tips (SuperSharp Enhanced, nanotools GmbH, Munich, Germany) with a nominal spring constant of 42 N/m and a nominal tip radius of 2 nm were used in tapping mode to obtain topographical images (Figure 3). Surfaces were scanned with a line rate of 0.7 Hz (control) and 0.5 Hz (predator exposed) and a resolution of 512 x 512 pixels^2^. A polynomial fit (degree 1) was subtracted from the image data to correct for the linear background. To reduce high frequency noise in the image, a low-pass filter (1.6 pointGaussian) was applied after the background correction. All processing and analysis of AFM images presented in this study was done using the JPK Data processing software (v 4.0.23). Only to calculate the particle size, unprocessed error images were imported into Adobe Photoshop Extended (v 12.0, Adobe Systems GmbH, Munich, Germany). To transfer the x-y calibration of the images to Photoshop, the Photoshop Extended measurement tool was used. Features were semi-automatically surrounded using the quick selection tool. Then the area of each feature was extracted using the measuring tool. Note that Photoshop was exclusively used to extract the feature areas and no further image processing was carried out. Three dimensional (3D) surface structural parameters were calculated according to the ISO 25178 using the JPK Data processing software. The following parameters were calculated for each topographical AFM image:

1) Average Roughness (S_a_)

$$S_{a}=\frac{1}{n}\underset{i}{\sum }\left|z_{i}\right|$$ (Eq. 2)

2) Peak-to-valley height (S_t_)

$$S_{t}=max_{i}z_{i}-min_{j}z_{j}$$ (Eq. 3)

Where *z*_*i*_ denotes the vertical deviation of point *i* from the mean plane and the sum includes all data points of the area (2.5 x 2.5 µm^2^ for our measurements). Both average roughness (S_a_) and peak-to-valley-height (S_t_) were calculated over the whole image after the baseline correction step.

### Measurement of the cuticle thickness

**Figure 4:**
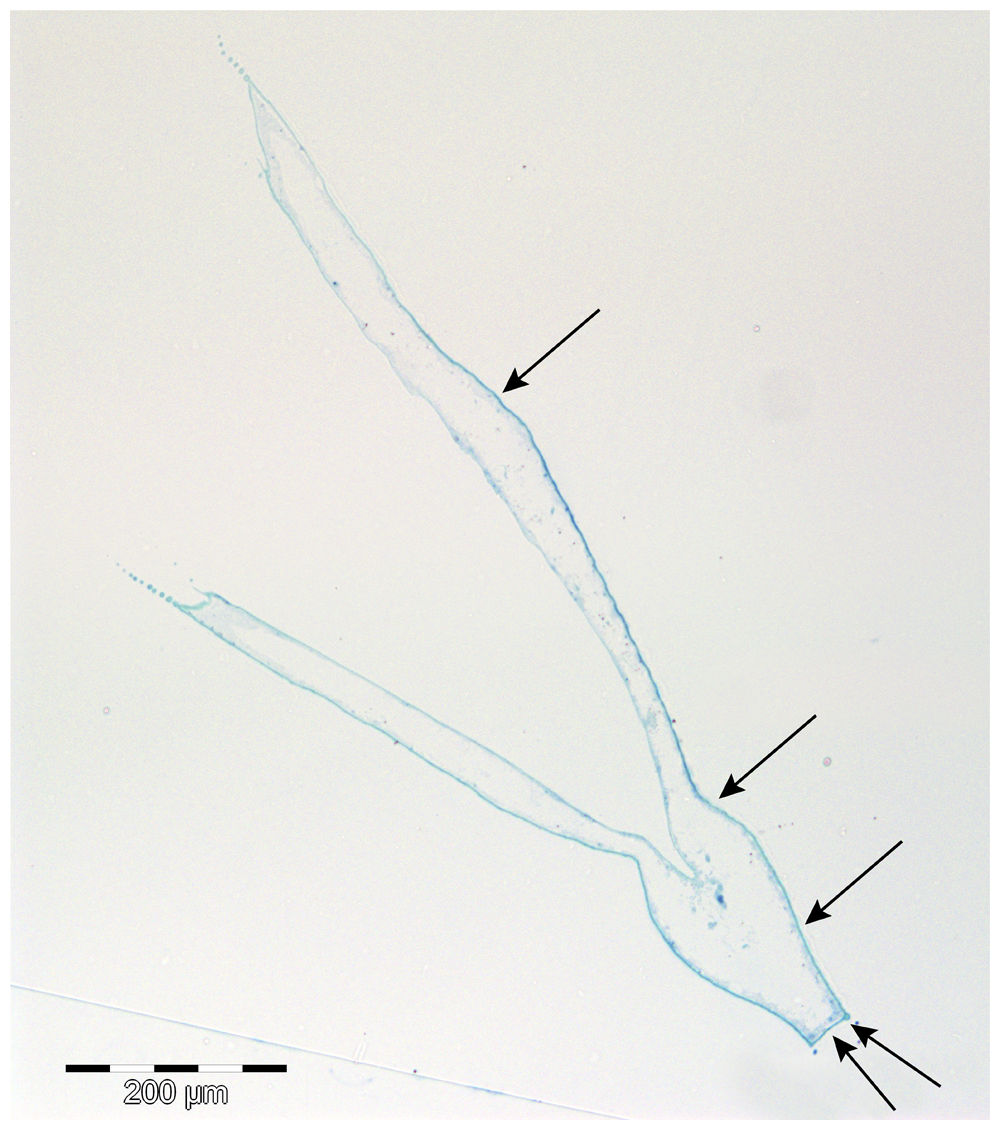
Location of the spots used for the measurement of cuticle thickness. Microscopic image of a 0.75 µm semithin section of an adult *D* *. magna* made in vertical alignment. The arrows indicate the spots which were selected for the thickness measurements.

**Figure 5:**
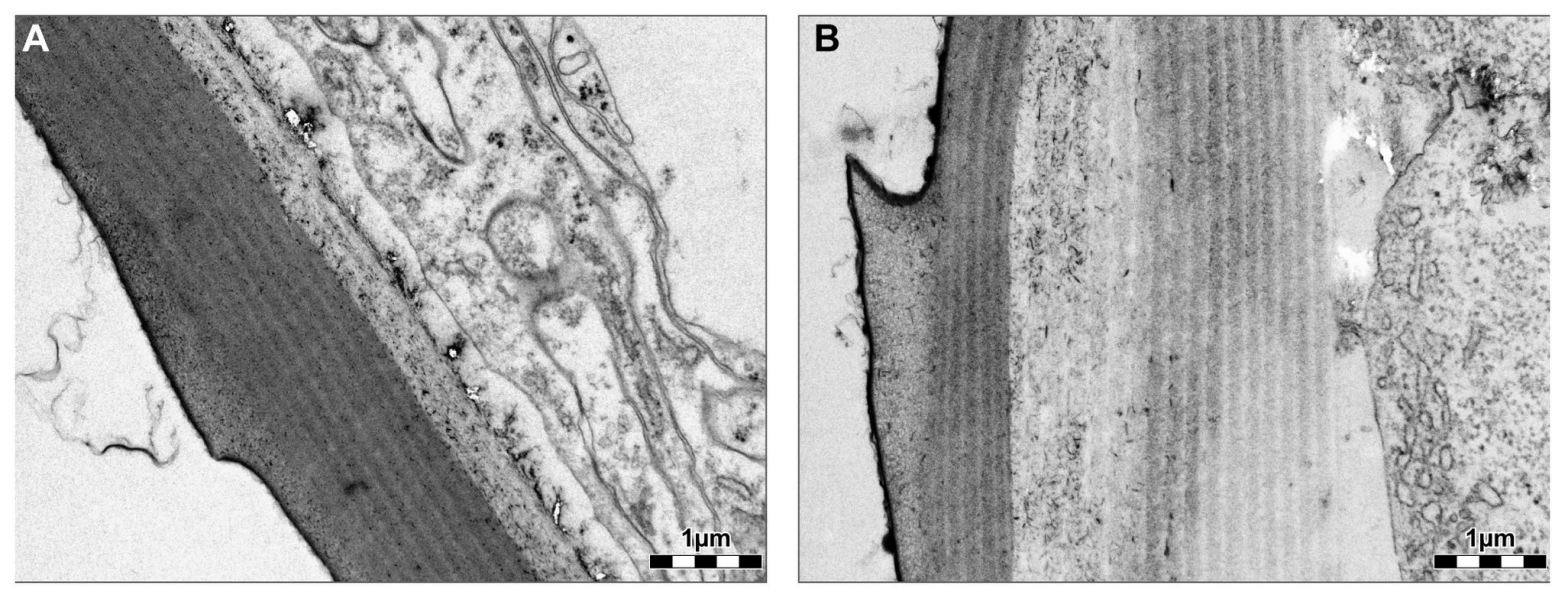
TEM micrographs of the outer cuticle layer in *D* *. magna* . Comparison of the outer cuticular layer of the carapace in a non-predator exposed (A) and a predator exposed (B) *D* *. magna* . Both micrographs were taken at 14,000x magnification.

 Three randomly chosen glutaraldehyde preserved daphnids per treatment were post-fixated in a 1% osmiumtetroxide solution. Then the daphnids were embedded in glycide ether 100 (Epon, Carl Roth GmbH & Co. KG, Karlsruhe, Germany) and 0.75µm semithin sections were made with a vertical alignment using an ultramicrotome (RMC MTXL, Boeckeler Instruments Inc., Tucson, USA). Semithin sections were stained using Richardson’s stain [53]. Cuticle thickness was measured using digital image analysis software (Cell^P, Olympus Deutschland GmbH, Hamburg, Germany) connected to a light microscope. For the analysis, one semithin section per *Daphnia* , approximately 140 µm craniad of the tail spine base, was chosen. Cuticle thickness was measured at 5 points of the outer cuticular layer of the carapace (Figure 4) and at each point, five measurements were taken. Additionally, ultrathin sections of approximately 60 nm thickness, obtained from the same region, were used to produce transmission electron microscope micrographs (Figure 5).

### Analysis of the pillar diameter

 The diameter of the pillars connecting the outer and the inner layer of the carapace [36,54] was compared between predator exposed and non-predator exposed *D* *. magna* . For that purpose the carapaces of five randomly chosen, ethanol preserved daphnids per replicate were dissected. Thereby, one cut was set posterior of the maxillary gland and the second cut was done close to the dorsal ridge to obtain two carapace halves. Additionally, one small cut was set at the ventral side of the carapace to allow mounting without deformation of the carapace. The dissected carapace parts were then mounted on a microscope slide using VECTASHIELD^®^ mounting medium (Vector Laboratories, Inc., Burlingame, USA). Images of the pillars were taken using a digital camera (Olympus DP 72, Olympus Deutschland GmbH, Hamburg, Germany) attached to a fluorescence microscope (Olympus BX 61, Olympus Deutschland GmbH, Hamburg, Germany). The pillars were depicted utilizing the autofluorescence of the carapace (GFP filter). For each *Daphnia* , the pillars within a 125 x 150 µm grid placed in the centre of each carapace sample were analysed. The average pillar diameter of each region of interest was analyzed using the particle analysis tool of the digital image analysis software Cell^P (Olympus Deutschland GmbH, Hamburg, Germany).

### Statistical analysis

 Statistical analyses were performed using the software package PASW Statistics 18 (SPSS Inc., Chicago, USA). Note that body length and –width of the daphnids have not been measured prior to the analysis of the focal traits to avoid any mechanical damage to the samples and thus the statistical analyses do not include an adjustment for possible size-dependencies. Alike, an adjustment for correlations between the focal traits was not possible because the chosen methods are invasive and require a different, incompatible treatment of the samples and hence data on all three focal traits could not be obtained from the same individual. Nevertheless, this does not affect the conclusions drawn from the results since individuals of the same developmental stage were compared to evaluate the biological relevance of the ultrastructural defences.

For each *Daphnia* analysed using AFM, a mean Young’s modulus was calculated. The data were tested for normality and homogeneity of variance and a log-transformation was applied to obtain homogeneity. A two-way ANOVA with treatment and replicate as fixed factors was performed to test for differences between predator exposed and non-predator exposed daphnids. The data obtained from the analysis of the pillar diameter were first used to calculate a mean pillar diameter for each *Daphnia* and the Mann–Whitney U test was performed due to violations of normality and homogeneity of variance. The results of the AFM-based surface analysis and the cuticle thickness measurements are presented graphically since a statistical analysis was not possible due to the small sample size. Values are expressed as the mean ± SE.

## Results

**Figure 6:**
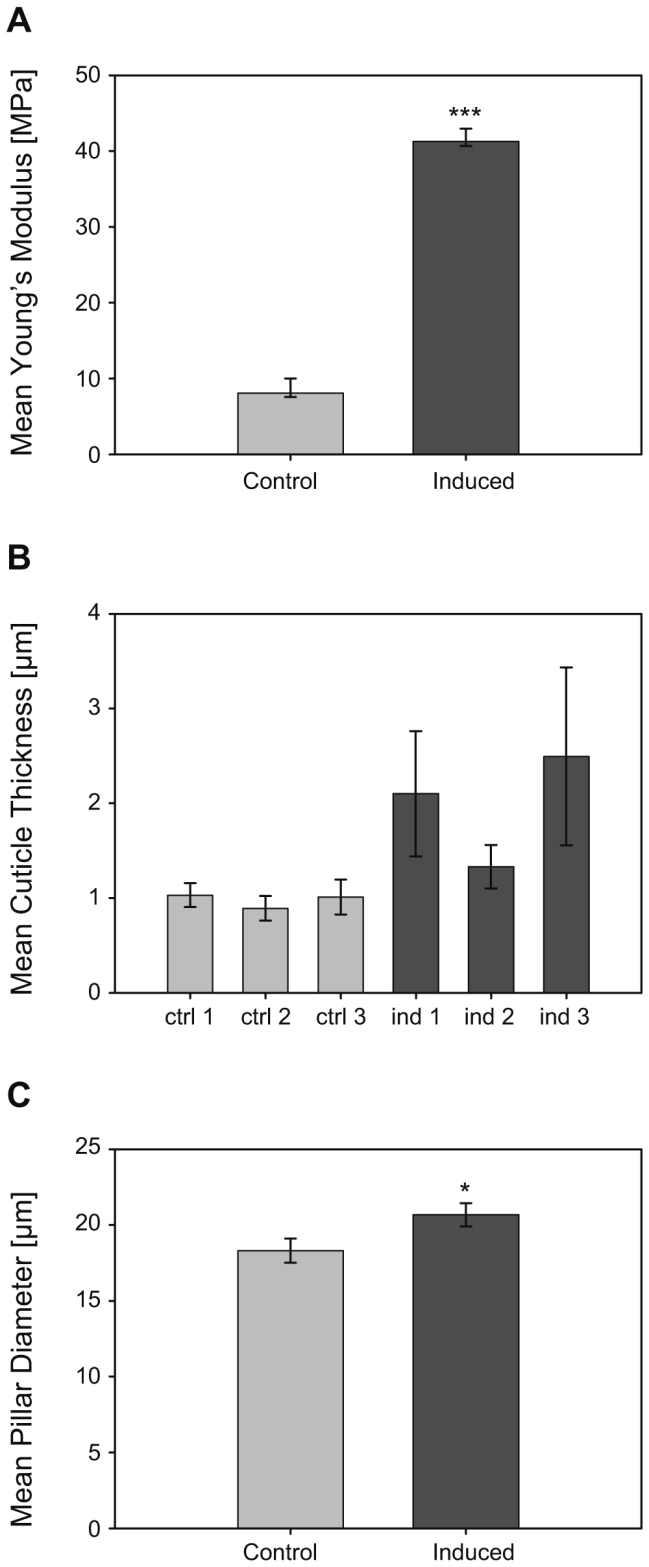
Comparison of the material properties of the carapace in predator- and non-predator exposed *D* *. magna* : A) comparison of the Young’s modulus, referring to cuticle hardness, measured using atomic force microscopy (non-predator exposed N = 15, predator exposed N = 15). The error bars show the back-transformed standard deviation (SD) of the log-transformed data used for analysis, the asterisks indicate the significance level (*** *P* < 0.001) based on a two-way ANOVA (F_1,24_ = 60.795). B) Comparison of the cuticle thickness measured using light microscopy. Mean cuticle for each measured individual is plotted, the error bars show the standard deviation (SD); and C) Comparison of the pillar diameter measured using fluorescence microscopy (non-predator exposed N = 14, predator exposed N = 12). The error bars show the standard error of mean (SE), the asterisks indicate the significance level (* *P* < 0.05) based on a Mann–Whitney U test (Z = -2,057).

 The AFM analysis of the cuticle properties revealed that the cuticle of *Triops*-exposed *D* *. magna* is significantly harder than the cuticle of control individuals: The two representative indentation curves (Figure 2) show that for identical indentation forces, the AFM tip is pushed much further into the cuticle of the control specimen (red curve), compared to the cuticle of the *Triops*-exposed specimen (blue curve). At a force of 10 nN, for example, the indentation depth on the non-predator exposed specimen is approximately 40 nm, while on the predator exposed specimen an indentation depth of only 15 nm is observed. Fitting the modified Hertz model (Equation 1) to all 1586 force vs. indentation curves showed an average Young’s modulus of 8.06 ± 4.92 MPa for the non-predator exposed and 41.28 ± 21.24 MPa for the predator exposed specimens. There was a significant main effect of the treatment on cuticle hardness (two-way ANOVA; F_1,24_ = 60.795; *P* < 0.001; Figure 6A) but no significant main effect of the replicate (two-way ANOVA; F_2,24_ = 1.785; *P* = 0.189) and no significant treatment x replicate interaction (two-way ANOVA; F_2,24_ = 0.193; *P* = 0.826).

As can be seen from the representative AFM images (Figure 3) and from the cross sectional profiles (Figure 2B and 2E), the increase in cuticle stiffness (Young’s modulus) is accompanied by smoother and more compact carapace surface morphology. The average surface roughness (S_a_) and the peak-to-valley height (S_t_) of the predator exposed specimen (Figure 2A and B) is reduced to S_a_ = 36.96 nm and S_t_ = 351.6 nm, compared to S_a_ = 50.22 nm and S_t_ = 481.8 nm for the control specimen. Furthermore, the mean particle size of surface corrugations increases from 0.05 ± 0.004 µm^2^ (n = 28) for the control specimen to 0.087 ± 0.007 µm^2^ (n=26) for the predator exposed animals. N represents the number of particles analysed within a single image. Note that because of the rather small radii of curvature in *D* *. magna* , and the limited z-range of the AFM scanner (15 µm in the imaging mode, compared to 100 µm in the force spectroscopy mode), only a small number of AFM images could be obtained for the carapace. The values of surface roughness and particle size were therefore extracted from only two representative images and thus a statistical analysis of these values was not possible.

Moreover, compared to control individuals, cuticle thickness seems to be considerably increased in predator exposed *D* *. magna* (Figure 6B): Cuticle thickness in predator-exposed individuals was approximately doubled in comparison to non-induced daphnids (3.95 ± 0.41 µm vs. 1.95 ± 0.08 µm). This result is supported by the TEM-micrographs (Figure 5), which indicate that the observed difference in cuticle thickness is predominantly due to amplification of the banded layer of the procuticle. Note that no statistical test could be performed due to the limited sample size. Finally, the mean diameter of the pillars which connect the outer and inner cuticular layers is significantly increased in predator-exposed daphnids (Mann–Whitney U test; Z = -2,057; *P* = 0,041; Figure 6C). Compared to non-predator exposed daphnids the mean pillar diameter of predator-exposed daphnids was approximately 10% enlarged.

## Discussion

 The results of our study indicate that the presence of *T* *. cancriformis* does not only induce large scale morphological defences in *D* *. magna* [37,38], but that these defences are accompanied by ultrastructural defences. By using AFM based nanoindentation, we could clearly show that the elastic modulus of the cuticle of *Triops*-exposed *D* *. magna* increases approximately five-fold. This increased elastic modulus is accompanied by a more compact and flatter surface morphology on the outer cuticle at the nano-scale. At the same time, optical microscopy of semithin sections and fluorescence microscopy of the carapace of *Triops*-exposed daphnids revealed a two-fold increase in cuticle thickness and a roughly 10% increase in pillar diameter, respectively. The measured increase in cuticle thickness is further supported by TEM-images depicting differences in the cuticle of predator-exposed compared to non-predator exposed daphnids. Namely, the banded layer of the procuticle is thickened, presumably due to an extended postmoult synthesis of cuticle material which was also observed in predator exposed *D* *. middendorffiana* and *D* *. pulex* [35].

Increased exoskeleton strength and thickness as a morphological defence in *Daphnia* has been discussed in a number of studies [21,24,34]. However, so far only few studies could actually demonstrate a predator-induced increase in the elastic properties of the carapace [35,36,55]. The increased elastic modulus renders the carapace of *D* *. magna* more rigid and together with the conspicuous morphological defences [37] it should provide more effective protection.

In the nanoindentation experiments we used sharp AFM tips (tip radius ≤15 nm) and limited the indentation depth to approximately 50 nm, i.e. we only probed the cuticle surface at the nanometre scale. The observed increase in the elastic modulus is therefore not caused by the increased cuticle thickness or other micrometer scale structural changes, such as the increased pillar diameter [56]. Hence, we can conclude that the predator-induced increase in cuticle thickness and pillar diameter acts as an additional structural defence mechanism in *D* *. magna* , which, together with the increased elastic modulus, increases the overall stiffness of the carapace. Possible explanations for the increased elastic modulus of the outer cuticle surface could be a different chemical composition of the cuticle, as well as a denser packing and/or a more compact nano-structure. The representative AFM images of the outer cuticle’s nanoscale topography (Figure 3) indicate that a more compact surface structure might indeed be one reason for the increased elastic modulus. However, further structural investigations at the nanometre scale, as well as studies of the chemical composition of the outer cuticle are necessary to resolve this question.

As already mentioned above, in addition to an increased elastic modulus and a thicker outer cuticle, we also found a *Triops*-induced increase in the diameter of the pillars connecting the outer and inner cuticle of the carapace in *Daphnia* . These structures are comparable to the so called “supporting epidermis” found in the branchiostegites of shrimp which are assumed to provide stability for the carapace [57]. A predator-induced increase in the pillar diameter has already been shown in *D* *. cucullata* exposed to *Chaoborus*-kairomones [36]. Together with their fibrous extensions, which probably provide additional stability, these pillars can be assumed to contribute to a higher overall stiffness of the exoskeleton in *Triops*-induced *D* *. magna* . The combination of a harder and thicker cuticle and the increased pillar diameter represents a sort of “lightweight” architecture which maximises stability while keeping the material expenditures relatively low [36]. Hence, this cost-saving strategy might be especially favourable in organisms which frequently moult. Since morphometric defences such as helmets cannot be developed within a single moult, the development of a stronger armour may act as a first-line defence. Furthermore, costs involved in forming this defence can be saved within a very short time frame if predation pressure is reduced in the environment. This may account for the frequently observed predator-induced plasticity in many arthropods, as reversibility of the protective trait is a prerequisite for the evolution of inducible defences.

The *Triops*-induced structural defences in *D* *. magna* have likely evolved over a long time scale of coexistence in their shared habitat, i.e. ephemeral waters. The mode of operation of these defences may thus be caused by the way *Triops* is feeding: When catching a prey item, the prey is first encaged by the endopodites and the spines of the endites and then it is taken into the midventral food groove and transported towards the mandibles [58]. In the food grove, the prey is processed by abduction-adduction movements of the spiky gnathobases while it is transported towards the maxillules and mandibles which finally crush the prey [58]. Given this mode of feeding, it seems likely that, in addition to the induced bulkiness of *D* *. magna* , a greater rigidity of the carapace should increase the prey’s chance of escape. By resisting deformation, and thus preventing a firm grip of the predator, a stiffer carapace may allow the prey to slip out of the feeding apparatus. Moreover, the combination of a harder and thicker cuticle should render the daphnids less susceptible to being pierced by the heavily sclerotised appendages of *Triops*. Hence, these ultrastructural defences should act synergistically with the previously shown bulkiness in *Triops*-induced *D* *. magna* [37,38]. Taken together, this array of defensive traits should act as a very effective protection against *Triops*-predation, as shown in previous predation trials [38].

Our findings support the idea that easily detectable shifts in morphometry may be accompanied by less conspicuous changes, such as an increase in cuticle strength in *Daphnia* [24]. This is concordant with previous studies in other *Daphnia* species, where a thicker cuticle [35] and a predator-induced increase in the elastic properties of the carapace have been demonstrated [36,55]. An increased exoskeleton thickness to reduce predator attack efficiency has been shown in a wide range of taxa, such as predator-exposed dragonfly larvae [15], aquatic snails [59,60] and bivalves [61] and seems to be a common strategy to increase fitness under predator stress.

To conclude, by applying an interdisciplinary approach between biology and nanoscience we could reveal that the *Triops*-induced large scale morphological defences in the clonal line of *D* *. magna* used for our experiments are accompanied by the expression of defensive traits in the ultrastructure of the carapace. Thereby, exoskeleton rigidity is considerably enhanced by an increased cuticle hardness, - thickness and size of the connective pillars. Increasing exoskeleton integrity should be an advantageous strategy when facing predators which have to grab and crush or suck their prey instead of ingesting it whole. Since many aquatic predators, especially invertebrates, fulfil these criteria, we assume that comparable structural defences should be widespread in both aquatic and terrestrial arthropods. This assumption is supported by a few studies that could reveal similar defence mechanisms in other *Daphnia* species [35,36,55]. However, further studies are necessary to assess the full spectrum and distribution of such defences across different predator–prey systems. This may help to understand the nature of inducible defences which evolve in a complex cost-benefit framework.
